# Surgical Aortic Valve Replacement and Renal Dysfunction: From Acute Kidney Injury to Chronic Disease

**DOI:** 10.3390/jcm13102933

**Published:** 2024-05-16

**Authors:** Antonio Lacquaniti, Fabrizio Ceresa, Susanna Campo, Antonella Smeriglio, Domenico Trombetta, Francesco Patanè, Paolo Monardo

**Affiliations:** 1 Nephrology and Dialysis Unit, Department of Internal Medicine, Papardo Hospital, 98158 Messina, Italy; 2 Cardiac Surgery Unit, Papardo Hospital, 98158 Messina, Italy; 3 Department of Chemical, Biological, Pharmaceutical and Environmental Sciences, University of Messina, 98122 Messina, Italy

**Keywords:** acute kidney injury, renal replacement therapy, surgical valve replacement (SAVR), renal recovery, AKI to CKD transition

## Abstract

**Background:** Surgical aortic valve replacement (SAVR) is often complicated by acute kidney injury (AKI). Identifying patients at risk of AKI is important to start nephroprotective strategies or renal replacement therapy (RRT). This study investigated the incidence and risk factors of post-operative AKI in SAVR patients. Chronic kidney disease (CKD) developed in the post-cardiac-surgery follow-up period was also assessed. **Methods**: A total of 462 SAVR patients were retrospectively enrolled. The primary endpoint was the occurrence rate of AKI after surgery. Kidney recovery, during two planned outpatient clinic nephrological visits within 12 months after the surgery, was assessed. **Results**: A total of 76 patients experienced an AKI event. A Kaplan–Meier analysis revealed that subjects with CKD stage IV had a time to progression of 2.7 days, compared to patients with stages I–II, who were characterized by the slowest progression time, >11.2 days. A Cox regression indicated that CKD stages predicted a higher risk of AKI independently of other variables. During their ICU stay, 23 patients died, representing 5% of the population, most of them requiring RRT during their ICU stay. A severe CKD before the surgery was closely related to perioperative mortality. During the follow-up period, 21 patients with AKI worsened their CKD stage. **Conclusions**: AKI represents a common complication for SAVR patients in the early post-operative period, prolonging their ICU stay, with negative effects on survival, especially if RRT was required. Pre-operative CKD >3 stage is an independent risk factor for AKI in patients undergoing SAVR.

## 1. Introduction

Surgical valve replacement (SAVR), a well-established treatment modality for symptomatic aortic valve disease, is often complicated by acute kidney injury (AKI), which occurs in the early post-operative period and significantly increases the risk of death [1].

Despite recent improvements to the early diagnosis and prevention post-operative AKI, its incidence after cardiac surgery is reported to remain as high as 30% [2].

Identifying patients at risk of AKI in their pre-operative period is extremely important to plan optimal and personalized therapies, and, if an AKI occurs in the post-operative period, achieve an optimal decision on whether to start nephroprotective strategies or a renal replacement therapy (RRT), improving the successful rate of these treatments.

However, scarce evidence refers to the prognostic factors of post-operative AKI.

**Figure 1:**
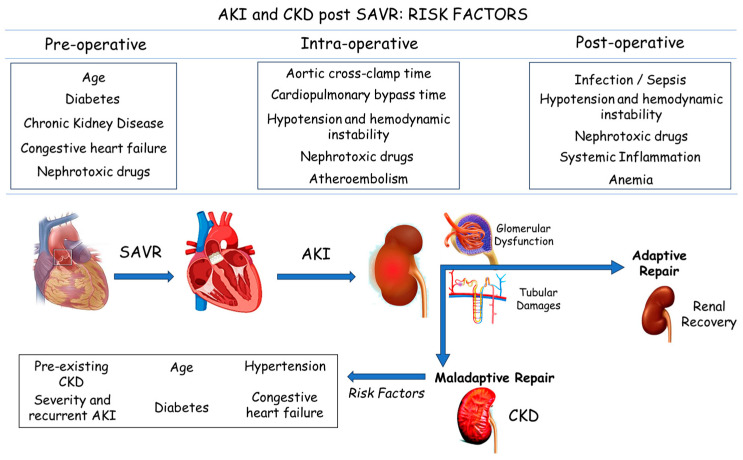
AKI and CKD post SAVR: the risk factors. Abbreviations: AKI: acute kidney injury; CKD: chronic kidney disease; SAVR: surgical aortic valve replacement.

It is known that high pre-operative creatinine levels, end-stage renal disease, comorbidities such as chronic heart failure or diabetes, the long duration of cardiac surgery, and an increased post-operative lactate level can be identified as classical predictors of peri-operative AKI in patients undergoing cardiac surgery [3,4] (Figure 1). 

For these reasons, a lack of consensus exists with regard to the definition of AKI in the post-surgery period. In 2019, a joint meeting of the Acute Disease Quality Initiative (ADQI-24) and the PeriOperative Quality Initiative (POQI-7) was convened to address post-operative AKI (PO), using a Delphi method to achieve consensus, but this was only valid for major non-cardiac surgery [5].

However, the recommendation of these experts was based on the application of the Kidney Disease Improving Global Outcomes (KDIGO) criteria for AKI occurring within 7 days of an operative intervention.

Generally, patients who undergo cardiac surgery could reveal in the early post-operative period only minor increases in serum creatinine, failing to meet the KDIGO criteria for a precocious AKI diagnosis and suggesting subclinical AKI, which has a higher risk of mortality and a longer hospital stay [6].

Chronic kidney disease (CKD) is associated with poorer outcomes after SAVR compared with patients with normal kidney function [7]. In fact, several patients with aortic stenosis, a degenerative disease closely related to aging, also have CKD, and the two interact with each other: a stenosis of the aortic valve induces renal hypoperfusion, while CKD worsens the aortic valve’s calcification and, consequently, the stenosis [8]. The full recovery or the partial resolution of these mechanisms could explain the increased glomerular filtration rate (GFR) following SAVR, especially in patients with the worst renal function [9,10,11]. In this context, the early differentiation of patients at higher risk for AKI onset is crucial, evaluating clinically useful biomarkers will lead to a precocious diagnosis of AKI and the start of rapid nephroprotective strategies, such as the maintenance of adequate blood pressure and the avoidance of volume depletion or nephrotoxic drugs. Recently, several new urinary biomarkers of kidney injury, such as the tissue inhibitor of metalloproteinases-2 × insulin-like growth factor-binding protein 7 (TIMP-2 × IGFBP7), have been identified for early diagnosis of AKI, anticipating a later diagnosis based on creatinine variations and the reduction of urinary output [12].

The other challenge is identifying the prognostic factors of kidney recovery (KR) and evaluating the mechanisms that could solve or worsen renal function, with no specific recommendations available for clinicians about the follow-up and management of patients with AKI post SAVR.

Starting from these assumptions, we retrospectively investigated the incidence and risk factors of post-operative AKI in patients who underwent SAVR, while also evaluating a subgroup of patients with endocarditis as the primary reason for cardiac surgery. The CKD developed in the post-cardiac-surgery follow-up period was also assessed.

## 2. Materials and Methods

### 2.1. Study Population

This retrospective cohort study was conducted on a group of consecutive patients undergoing SAVR due to aortic valvular heart disease. From 1 January 2017 to 31 December 2021, we identified 462 patients undergoing a first-time SAVR procedure at the Cardiac Surgery Unit of the Papardo Hospital, Messina, Italy.

All SAVR procedures were performed through a full or upper partial median sternotomy under moderate hypothermia and cold cardioplegic arrest.

Each procedure was performed with the use of a cardiopulmonary bypass (CPB) and an aortic cross-clamp (ACC) applied.

The surgeons decided the between a biological or mechanical valve according to the patient’s comorbidities.

All patients were transferred to the intensive care unit (ICU) after the operation. In all patients, the management of their anesthesiologists and surgeons was regular, with surgical procedures performed without complication.

Patients with multiple valve replacements, end-stage renal disease (ESRD) (CKD stage 5 or chronic dialysis use), or a coronary artery bypass were excluded from this study. Re-operations were also excluded. Patients treated with a transcatheter aortic valve implantation (TAVI) have been excluded from enrolment.

Patients who underwent SAVR due to endocarditis were analyzed as a different group from the rest of the cohort.

### 2.2. Study Variables and Outcomes

The pre-operative glomerular filtration rate, using the last creatinine measurement before surgery (within the range of zero to six days pre-op), was estimated (eGFR) using the Chronic Kidney Disease Epidemiology Collaboration (CKD-EPI) equation formula, and presented as categorial variables (eGFR ≥ 90, eGFR 60–89, eGFR 30–59, eGFR < 30) [13,14].

The primary endpoint of the study was the occurrence rate of AKI within seven days after surgery, which was defined according to the KDIGO definition. Serum creatinine and urinary output were used as the diagnostic standards of AKI. According to this classification, if the serum creatinine increases by ≥0.3 mg/dL within 48 h, the serum creatinine is 50% higher than the baseline within first seven days, or the urine output is below 0.5 mL/kg/hour for six hours, the patient is considered to have an AKI [15].

Patients beginning first-time dialysis treatment after their SAVR were categorized in the AKI group.

Moreover, among patients with AKI, we assessed the RRT incidences during their ICU stays, and their kidney recovery during a planned outpatient clinic nephrological visit, using the lowest measured creatinine from 2 periods: 90–180 days after the surgery and at the next nephrological visit, scheduled after 9–12 months.

According to the Acute Disease Quality Initiative consensus document, kidney recovery was defined as (1) complete kidney recovery (defined as creatinine < 1.15 × baseline creatinine), (2) partial kidney recovery (defined as creatinine < 1.5 × baseline creatinine), (3) failure to recover (defined as creatinine > 1.5 × baseline creatinine), or (4) ongoing dialysis treatment [16].

Only patients with AKI who had a recorded creatinine sample during the specified time periods and survived the entire duration were considered in these particular analyses.

### 2.3. Statistical Analyses

Statistical analyses were performed with NCSS for Windows (version 4.0), Med-Calc (version 20.115; MedCalc Software Acacialaan, Ostend, Belgium) software, and the GraphPad Prism (version 9.4.1; GraphPad Software, Inc., San Diego, CA, USA) package.

Baseline characteristics were reported as frequencies, with percentages for categorical variables and medians with interquartile ranges for continuous variables.

Kaplan–Meier curves were generated to assess the progression to the endpoint, defined as a diagnosis of AKI during their ICU stay in subjects with different stages of CKD. In particular, we analyzed three groups: CKD stage I and II, with a GFR between 90 and 60 mL/min; CKD stage III, with a GFR between 60 and 30 mL/min; and CKD stage IV with a GFR between 30 and 15 mL/min. Differences were evaluated using the log-rank test.

Adjusted risk estimates for the progression to the endpoint, defined as the onset of AKI during the ICU stay after SAVR, were calculated using a univariate followed by multivariate Cox proportional hazard regression analysis. The results were reported with hazard ratios (HRs) and 95% CIs, and the level of statistical significance was recognized as a *p*-value < 0.05.

This study was approved by the local ethical committee of the University of Messina (Protocol number 96-23; 19 December 2023). Each patient recruited for the study gave written consent before their enrolment at the time of their admission inro the cardiac surgery ward, and before their SAVR.

## 3. Results

### 3.1. Baseline Characteristics

The demographic and clinical characteristics of the study population are presented in Table 1.

The entire cohort consisted of 462 patients (244 males [53%]), with a mean age of 66.2 ± 11.4 years. The main comorbidities were represented by diabetes, dyslipidemia, and smoking, which affected almost one third of the cohort. Additionally, almost all patients received antihypertensive drugs. In particular, all hypertensive patients were treated by Angiotensin-converting enzyme inhibitors/angiotensin 2 receptor blockers, whereas beta blockers were administered in 65% of patients. Moreover, 40% of all patients were affected by hyperlipidemia, which was treated with statins. No differences between groups of treatment were assessed and no correlations were found with the study endpoints.

In our cohort, 17% (*n*: 78) of treated patients suffered from CKD before their surgical treatment, whereas kidney function was normal in the remaining patients (*n*: 384, 83%). In particular, 33 out of 78 (42%) patients had CKD stage I–II, 29 patients (37%) had CKD stage III, and the remaining 16 subjects had CKD stage IV (21). The mean aortic cross-clamp time was 104 ± 42 min, whereas the mean value of the cardiopulmonary bypass time was 138 ± 57 min. All patients were transferred to the ICU after the surgery, with a mean length of stay of 11.4 ± 7.9 days (median 10 days; IQR: 8–13 days).

### 3.2. AKI Prevalence and Dialysis Treatment

A total of 76 patients (16.4%) experienced an AKI event during their stay in the ICU. The AKI group was older, with more comorbidities, such as diabetes and hyperlipidemia, compared to the No-AKI group. CKD’s prevalence was another difference between the two groups, involving most of them (67%), especially if a low eGFR was found. Moreover, the AKI group was related to a longer aortic cross-clamp time (127 ± 31 vs. 91 ± 27; *p* < 0.05) and cardiopulmonary bypass time (151 ± 19 vs. 101 ± 29; *p* < 0.05) compared to the time observed for the No-AKI group. Furthermore, the onset of AKI was closely related to a longer LOS in the ICU, when compared to the No-AKI group (13.6 ± 4.9 vs. 3.8 ± 1.4 days; *p* < 0.05).

The Kaplan–Meier analysis revealed that subjects with CKD stage IV experienced a significantly faster evolution to the endpoint (*p* < 0.001), with a mean follow-up time to progression of 2.7 days (95% CI, 1.2–4.9), compared to patients with stages I–II, who were characterized by the slowest progression time, >11.2 days (95% CI, 5.2–16.3). To identify the putative risk factors associated with AKI, we performed a Cox regression analysis, inserting into the model all variables that were different at the enrollment in patients who had an AKI or not (age, hyperlipidemia, smoking, diabetes, CKD stages). We also inserted into the model the aortic cross-clamp and cardiopulmonary bypass times as not negligible risk factors for AKI.

The univariate analysis showed that age (HR 1.06; 95% CI, 1.02 to 1.07; *p*: 0.04), diabetes (HR 1.05; 95% CI, 1.02 to 1.09; *p*: 0.03), CKD stages (HR 1.13; 95% CI, 1.04 to 1.22; *p*: 0.002), aortic cross-clamp time (HR 1.09; 95% CI, 1.05 to 1.16; *p*: 0.001), and cardiopulmonary bypass time (HR 1.08; 95% CI, 1.08 to 1.22; *p*: 0.004) were significantly associated with the endpoint, whereas other comorbidities, such as hyperlipidemia and smoking, failed to reach statistical significance.

A multiple Cox regression was constructed, simultaneously inserting into the model all of the variables found to be significantly associated with the endpoint in the univariate analysis (age, diabetes, CKD stages, aortic cross-clamp time, and cardiopulmonary bypass time). The results from this analysis indicated that CKD stages predicted a higher risk of AKI independently of other variables, such as the aortic cross-clamp and cardiopulmonary bypass times. In detail, the hazard ratio for AKI increased gradually and significantly with increasing kidney failure severity. A decrease in GFR of 10 mL/min was associated with a 10% increased risk of AKI (HR 1.10; 95% CI, 1.04 to 1.25; *p* 0.0004), while stage IV CKD represented the highest risk for the studied cohort developing an AKI event. Table 2 summarizes the Cox analysis.

### 3.3. RRT, Kidney Recovery, and All-Cause Death

A total of 76 patients underwent SAVR and developed an AKI during their ICU stay, and 32 (42%) of them were treated with RRT. In particular, all patients received a continuous veno-venous hemodialysis treatment, with a mean time of therapy of 4.1 ± 3.1 days. In 12 patients, the therapy was suspended due to kidney function improvement, whereas the remaining 20 patients continued the dialysis treatment during their ICU stay, until discharge or death.

After their ICU discharge, 45 patients started a follow-up period with planned nephrological visits to evaluate the new onset of CKD or a worsening of their renal function, as the patients had a reduced eGFR at enrolment.

All patients completed the entire follow-up period. Among the patients with pre-existing CKD, 24 subjects, of whom 8 belonged to stage I–II and 16 belonged to stage III, had no change in renal function, whereas the remaining 21 experienced worsened renal function. In particular, 10 out of 16 patients with CKD stage IV required chronic hemodialysis for the 1 year after their SAVR procedure, as did 7 patients with CKD stage III. This interaction between baseline renal function and the development of AKI was highlighted by grouping the cohort into three tertiles of renal function: GFR ≤ 30, GFR 31–60, and GFR over 60, revealing that kidney recovery increased in a linear manner with the highest GFR tertile (*p* = 0.03).

During their ICU stay, 23 patients died, representing 5% of the entire studied population.

A total of 19 out of the 23 (82%) patients who died required RRT during their ICU stay, and having severe CKD before the surgery, defined as stage IV, was closely related to perioperative mortality.

### 3.4. Endocarditis and AKI

As underlined in the methods section, separately from the studied population, we also evaluated 15 patients who underwent SAVR due to endocarditis. The mean age of this group was younger than the studied cohort (53.4 ± 7.2 vs. 66.2 ± 11.4 years), with a similar gender distribution. All these patients met the sepsis criteria with a high incidence of AKI in the post-cardiac-surgery period (12 out of 15 patients). In total, 9 out of 15 required RRT therapy and 7 (47%) of them died during their ICU stay.

## 4. Discussion

This study revealed that AKI represents a common complication for SAVR patients in the early post-operative period, prolonging their ICU stay, with negative effects on survival, especially if RRT was required.

The correlation between poor pre-operative renal function and worse long-term survival post SAVR is well documented [17].

However, although pre-operative renal function may be associated with post-operative AKI, further studies are required to select the optimal surgical procedure for preexisting renal dysfunction and to optimize and personalize nephroprotective strategies.

TAVI is the treatment of choice for patients with severe aortic stenosis and a high surgical risk due to advanced age and comorbidities, but the relationship between AKI, CKD risk, and the outcomes after TAVI is still unclear [18,19,20]. TAVI requires a contrast agent’s administration with an increased risk of contrast-induced nephropathy and the distal embolization of atherosclerotic debris to the renal vascular bed, secondary to the use of catheters in the aorta. Moreover, hypotensive episodes could induce prerenal dysfunction, leading to renal hypoperfusion. Thus, AKI after TAVI is the final effects of prerenal damages associated with the acute tubular injury induced by nephrotoxic drugs, i.e., contrast agents [21].

Large registries are essential to confirm whether TAVI could induce fewer nephrotoxic effects than SAVR in a real-world setting. If the surgical approach is the choice for treating the valve disease, the CPB and the ACC times are potential modifiable risk factors, but relatively less is known about their relationship with AKI over the post-operative period. A longer CPB, altering the renal blood flow, is independently associated with precocious AKI, considering that the longer the CPB time, the greater the time of contact between the patient’s blood and the foreign surface of the extracorporeal circuit, and then the greater the severity of Systemic inflammation and coagulopathy [22,23,24].

The prolonged ACC time significantly correlated with worse clinical outcomes, including in-hospital mortality, prolonged hospitalization, prolonged ventilation, and renal complications, as consequences of ischemic processes and embolization involving the heart, brain, and the kidneys [25]. Our data confirm this evidence, revealing the close and independent connections between these two technical parameters and AKI after SAVR.

Interestingly, in addition to its early effects on renal function, a longer ACC time was independently associated with decreased late survival, defined as 25 years of follow-up, whereas a longer CPB time was not an independent predictor of decreased late survival [26].

Longer cross-clamp times are associated with a greater risk of myocardial ischemia, and this effect should also be evaluated to analyze in depth the potential link between prolonged ACC times and a new onset of CKD after SAVR [27].

In fact, we demonstrated that this population had a not-negligible risk of developing a renal disease or worsening their already altered renal dysfunction after an AKI event, revealing that kidney recovery increased in a linear manner with GFR values, and suggesting that nephrological visits must be included in the follow-up programs of patients discharged after SAVR for detecting a kidney injury leading to CKD. Thus, cardiac surgery contributes to the CKD burden, especially because many patients have preexisting risk factors such as hypertension and diabetes. In this context, several biomarkers of injury, inflammation, or repair were assessed in settings of AKI transitioning toward CKD [28,29,30].

High levels of urinary urine epidermal growth factor (EGF) and monocyte chemoattractant protein-1 (MCP-1) were independently associated with a lower risk of a composite CKD outcome, whereas higher post-operative levels of urinary MCP-1 were associated with higher risk after a coronary artery bypass and/or valvular surgery, reflecting the ability of tubular cells to recover their function after an acute injury and stress [31].

AKI events, associated with the hemodynamic stress induced by hypotension or a prolonged ACC time, could determine late cardiac dysfunction and, consequently, the development of a cardio-renal syndrome (CRS). In its early phases, a type 1 CRS could be the physiopathological process inducing AKI, but, later, a chronic heart disease could cause a CKD (type 2 CRS). At the same time, we also revealed that a preexisting CKD > III stage was an independent major risk factor for AKI in patients undergoing SAVR, with consequent effects on their ICU LOS and mortality. This datum is not negligible, highlighting that aortic valve disease is a not rare condition in patients with CKD, with a prevalence of about 10%, higher than the 3.5% found in the general population [32].

Our data corroborate this close link between aortic valve disease and renal failure, considering that we enrolled 17% of the surgically treated patients who suffered from CKD. This trend is confirmed by other reports, underlying the wide ranges of CKD’s prevalence in patients treated with SAVR procedures [33]. In CKD, numerous metabolic contributors are involved in the development of degenerative valvular lesions, but, probably, secondary hyperparathyroidism related to hyperphosphatemia and an elevated phosphate/calcium product primarily induced valvular calcification [34,35]. In these patients, renal disease could represent the first step to inducing a chronic cardiac disease secondary to a valve dysfunction (type 4 CRS), which will indicate the patient to SAVR many years later.

Finally, we revealed a 5% rate of death during the ICU stay, with a high prevalence in patients requiring RRT and with a severe CKD before their surgery, leading to speculation about their relationship with peri-operative mortality, but not supported by our statistical analyses due the reduced number of patients. This is one of several limitations that should be recognized for this study. The cohort of AKI patients was relatively small and this condition did not allow for further statistical analyses, both during their ICU stay and during the follow up period, such as of the survival rate or cardiovascular events. Moreover, this was a retrospective observational study conducted at a single center and it should be interpreted as such, due to the inherent errors associated with this methodology. However, the single-center design guarantees the uniformity of the data collection and surgical technique. Finally, this study focused specifically on SAVR, and the findings may not be applicable to other valve procedures, such as TAVI or mitral valve surgery. Prospective cohort studies are needed to evaluate the personalized surgical approach and preventive strategies, starting from the assessment of presurgical kidney function in this study based on an estimated formula for GFR.

Despite the use of a multivariable Cox regression to establish the risk factors for AKI, some confounding factors might not have been accounted for, such as medication use or echocardiographic features.

The etiology of the aortic disease was not evaluated, underlined by the fact that aortic valve endocarditis was considered an exclusion criterion, but our data reported a high risk for AKI, RRT, and mortality in, separate from this study, patients with endocarditis, encouraging further studies to selectively enroll this high-risk population. In this context, which is closely related to sepsis, RRT could play important role after AKI stage, using adsorber filters as a therapeutical strategy to counteract the sepsis [36,37].

## 5. Conclusions

SAVR patients had high risk of AKI in the early post-operative period, with a prolonged ICU stay and negative effects on survival, especially if RRT was required. Moreover, these patients could have a not negligible risk of developing chronic renal disease or worsening their already altered renal dysfunction after an AKI event, suggesting that nephrological visits must be included in their follow-up programs after their discharge.

## Figures and Tables

**Table 1:** Characteristics of the study population.

	All Patients (*n*: 462)	AKI Group (*n*: 76)	No-AKI Group (*n*: 386)	*p*
Age, mean ± SD	66.2 ±11.4	69.4 ± 7.5	54.6 ± 4.9	<0.05
Male, *n* (%)	244 (53)	41 (54)	203 (52)	>0.05
Female, *n* (%)	218 (47)	35 (46)	183 (47)	>0.05
Hypertension, *n* (%)	405 (88)	73 (96)	332 (86)	>0.05
Hyperlipidemia, *n* (%)	195 (42)	60 (78)	135 (35)	<0.05
Smoking, *n* (%)	131 (28)	62 (81)	69 (18)	<0.05
Diabetes, *n* (%)	132 (29)	59 (77)	73 (19)	<0.05
CKD, *n* (%)	78 (17)	51 (67)	27 (4)	<0.05
Stage I–II	33	8	25	<0.05
Stage III	29	27	2	<0.05
Stage IV	16	16	-	<0.05
Aortic cross-clamp time, min	101 ± 49	127 ± 31	91 ± 27	<0.05
Cardiopulmonary bypass time, min	126 ± 43	151 ±19	101 ± 29	<0.05
RRT, *n* (%)	32 (7)	32 (42)	-	-
LOS in the ICU days, *n* (%)	11.4 ± 7.9	13.6 ± 4.9	3.8 ± 1.4	<0.05
Death, *n* (%)	23 (5)	21 (28)	2 (0.5)	<0.05

Abbreviations: CKD: chronic kidney disease; RR: renal replacement therapy; LOS: length of stay; ICU: intensive care unit.

**Table 2:** Univariate and multivariate Cox proportional hazards regression model for the incidence of acute kidney injury during the ICU stay.

	Univariate Analysis			Multivariate Analysis		
	HR	95% CI	*p* Value	HR	95% CI	*p* Value
Hyperlipidemia	1.07	0.94–1.12	0.27			
Age	1.06	1.02–1.07	0.04	1.03	0.97–1.04	0.08
Diabetes	1.05	1.02–1.09	0.03	1.02	0.99–1.04	0.06
Smoking	1.06	0.97–1.18	0.37			
CKD	1.13	1.04–1.22	0.002	1.10	1.04–1.25	0.0004
CKD I–II	1.03	1.01–1.05	0.04	1.02	0.97–1.03	0.07
CKD III	1.07	1.02–1.11	0.02	1.06	1.04–1.09	0.03
CKD IV	1.10	1.02–1.14	0.01	1.09	1.01–1.11	0.01
Aortic cross-clamp time	1.09	1.05–1.16	0.001	1.04	1.02–1.09	0.03
cardiopulmonary bypass time	1.08	1.08–1.22	0.004	1.08	1.03–1.12	0.02

Abbreviations: ICU: intensive care unit; CKD: chronic kidney disease; I–II: estimated glomerular filtration rate ranging from 90 and 60 mL/min; III: estimated glomerular filtration rate ranging from 60 and 30 mL/min; IV: estimated glomerular filtration rate ranging from 30 and 15 mL/min.

## Data Availability

The data underlying this article will be shared upon reasonable request to the corresponding author.
